# Isolation and Transcriptome Analysis of Phenol-Degrading Bacterium From Carbon–Sand Filters in a Full-Scale Drinking Water Treatment Plant

**DOI:** 10.3389/fmicb.2018.02162

**Published:** 2018-09-21

**Authors:** Qihui Gu, Qingping Wu, Jumei Zhang, Weipeng Guo, Yu Ding, Juan Wang, Huiqing Wu, Ming Sun, Luanfeng Hou, Xianhu Wei, Youxiong Zhang

**Affiliations:** State Key Laboratory of Applied Microbiology Southern China, Guangdong Provincial Key Laboratory of Microbial Culture Collection and Application, Guangdong Open Laboratory of Applied Microbiology, Guangdong Institute of Microbiology, Guangzhou, China

**Keywords:** microbial community structure, high-throughput sequencing, *Rhodococcus* sp. CS-1, polyhedral hollow polypropylene balls, transcriptome

## Abstract

Phenol is a typical organic contaminant in the environment. To date, the biodegradation of phenol by microorganisms remains the preferred method for its removal and remediation, but data on phenol removal by drinking water biofilters are lacking. In this study, we used high-throughput sequencing to investigate the microbial community structure in a carbon–sand biofilter. The results indicated that the predominant bacterial group was *Bacilli*, followed by *Gammaproteobacteria*, *Clostridia*, and *Alphaproteobacteria*. In addition, a strain was capable of degrading phenol at low concentrations of 500 μg/L within 100 min was isolated and identified as *Rhodococcus* sp. CS-1. Transcriptome analysis results showed that *Rhodococcus* sp. CS-1 was able to degrade phenol via both the catechol and protocatechuate branch of the β-ketoadipate pathway. Furthermore, some novel candidate biomarkers (copper oxidase, copper chaperone, and MarR/DeoR/TetR family transcriptional regulators) were successfully identified to be potentially involved in phenol biodegradation. This study indicates that carbon–sand filters have the potential for remediation of phenol. The application of native microorganisms to drinking water treatment system is an adaptive strategy in oligotrophic water environments.

## Introduction

Phenol is an environmental pollutant that is widespread due to its broad application in industrial processes, including oil refineries and chemical plants (Pradhan and Ingle, 2007). It can be found in groundwater (Ehrlich et al., 1982; Flyvbjerg et al., 1993) and even in drinking water (Jarvis et al., 1985). Even relatively low concentrations of phenol can affect aquatic life and pose a danger to humans (Loh et al., 2000). Thus, a method for the effective removal of low concentration phenol from drinking water souces is highly desired. To date, methods of phenol removal have been widely studied, including ozonation, activated carbon adsorption, and chemical oxidation. However, these processes are usually complex, expensive, and may even lead to secondary pollution. In addition, these methods are not effective in removing low concentrations of phenol. Biodegradation was proved to be an economic, environmental, and effective methods to remove low concentraton of phenol (Mordocco et al., 1999). To our knowledge, indigenous bacteria are well applied for *in situ* remediation (Jiao et al., 2011). Because these bacteria had good environmental adaptability, they could survive and increase contaminant remediation (Gentry et al., 2004). With respect to phenol contaminated drinking water source, isolating indigenous bacteria from drinking water treatment system to remove phenol is a good menthod. To our knowledge, more than 95% of waterworks in China continue to use conventional water treatment processes, including coagulation–flocculation, sedimentation, filtration, and disinfection, to purify drinking water (Hou et al., 2018). In addition, carbon–sand filters were common in drinking water plants in China today, it has been proved that organic matter and ammonium could be effectively removed by carbon–sand filters (Feng et al., 2013). Therefore, identification of microbial structure in carbon–sand filters could aid in the knowledge of the biodegradation capacity of these systems, and in the knowledge of whether there were microorganisms that could degrade target pollutant (Feng et al., 2013). Despite there have been some studies on carbon–sand filters microbial communities by metagenome analysis (Feng et al., 2013; Hou et al., 2018). The structure of microbial community in different sites was usually distinguishing. Because microbial communities in natural aquatic habitats are very sensitive to environmental perturbations (Paerl et al., 2010). Microbial community composition might be related to many environmental factors, including dissolved oxygen (DO), temperature, nutrients, and organic matter (Feng et al., 2013). To date, microorganism community structure in carbon–sand filters in Guangdong province, southern China was very limited. Therefore, it is necessary for us to analyze the microbial community composition in the carbon–sand filter where we sampled, and which would be a good guide for isolating functional bacteria.

Despite the significance of *Rhodococcus* strains as efficient phenol degraders in biotechnological processes (Pai et al., 1995; Margesin et al., 2005), phenol catabolism-related genes and its genetic regulation mechanism in rhodococci were still scarce, especially which were isolated from oligotrophic and micro-pollution environment. In previous studies, majority of phenol-degrading *Rhodococcus* strains were isolated from soil (Jones et al., 2004), sediments (Paisio et al., 2012), and wastewater (Arutchelvan et al., 2005) heavily contaminated with organic pollutants. Therefore, these bacteria always have a natural resistance to contaminants. In addition, the mechanism of phenol catabolism was often studied for these bacteria. However, research on how *Rhodococcus* strains isolated from oligotrophic and micro-pollution environment response to phenol is unknown, especially the reasons for variations in the level of expression of important enzymes and novel regulatory factors during phenol catabolism. To the best of our knowledge, transcriptomic technologies could improve our knowledge of global transcriptomic responses under different conditions, and it is a good tool to find some candidate biomarkers. For example, Liao et al. (2018) reported the global transcriptional response of *Citrobacter* sp. to 2,4,6-trinitrotoluene. Yi et al. (2015) revealed the tolerance and detoxification mechanisms of *Zymomonas mobilis* ZM4 to phenolic aldehyde inhibitor. Yoneda et al. (2016), elucidated the adaptive phenol tolerance and utilization in lipid-accumulating *Rhodococcus opacus* PD630 by comparative transcriptomics. Therefore, transcriptome is a promising approach to reveal the global genes expression in *Rhodococcus* during phenol biodegradation process.

The immobilization of microbial cells has garnered increasing interest in the field of pollutant degradation. Previous researchers have studied immobilized cells in terms of the biodegradation of phenol (Jiang et al., 2013), naphthalene (Manohar et al., 2001), pyrene (Xu et al., 2016), and hydrocarbons (Nie et al., 2016). In addition, this technology is usually applied in the pre-treatment and filtration processes in drinking water treatment plants (DWTPs). For example, the ozone-granular-activated carbon process is a common immobilization technology used in drinking water treatment that has performed well until now (Kainulainen et al., 1995). Immobilized cell systems have the potential to degrade toxic chemicals faster than planktonic cell systems, since they involve high densities of specialized microorganisms and it is good for recycling. As we known, there are not much immobilized material used in drinking water treatment system because of the safety. In China, polyhedral hollow polypropylene balls have been usually applied to immobilize microorganisms in drinking water pretreatment. But related reports about the immobilization of pure functional bacteria on polyhedral hollow polypropylene balls are limited. Majority of studies mainly focused on investigating microoganisms immobilized on granular active carbon (Gibert et al., 2013). Therefore, investigation of the biofilm-forming ability on polyhedral hollow polypropylene balls can evaluate the practical applicability of the immobilized functional bacteria cells in drinking water source bioremediation.

In this study, we investigated the microbial community structure in the sand–carbon filter in a DWTP using high-throughput sequencing. A strain capable of effectively degrading phenol at low concentrations was isolated and characterized. Moreover, the mechanism of phenol metabolism in this strain was investigated by comparative transcriptomics, and the biofilm development of the phenol degrader was also characterized. Furthermore, phenol degradation by immobilized cells in a continuous reactor was assessed.

## Materials and Methods

### Sampling, PCR Amplification, and Illumina MiSeq Sequencing

Two carbon–sand samples were obtained from a carbon–sand filter in a DWTP (surface river water as source water) in Guangzhou, South China. The source water contained approximately 6.0 mg/L DO; 0.13 mg/L ammonia (NH_3_-N); 2.13 mg/L chemical oxygen demand (COD); and 0.29 total chlorine with a turbidity of 46.2 NTU, pH of 7.39, and total hardness of 95. The drinking water treatment process of the DWTP included pre-chlorination, coagulation and sedimentation, dual-media filtration, and disinfection (with chlorine). Specifically, there is a 30-cm depth of granular-activated carbon (GAC), with a 70-cm layer of quartz sand on the bottom of the dual-media filter. Samples BAC0 and BAC10 were collected from the carbon–sand filter at depths of 0 and 10 cm, respectively. Three replicate samples were taken for samples BAC0 and BAC10. Briefly, carbon–sand samples were harvested from the four corners and the center point of each carbon–sand filter at the corresponding depth and then carefully collected into a sterile bag to produce one composite sample for each depth.

In order to investigate the microbial community in each carbon–sand layer, total genomic DNA was extracted from one carbon–sand samples using a soil DNA extraction kit (Sangon Biotech, Shanghai, China). The 16S rRNA gene was amplified using the primers 515 F (5′-GTGCCAAGCMGCCGCGGTAA-3′) and 806R (5′-GGACTACHVGGGTWTCTAAT-3′) with different barcodes for each sample (Ma et al., 2015). PCR amplification was performed as described previously (Bautista-de los Santos et al., 2016). PCR conditions were as follows: 95°C for 3 min; 27 cycles of 95°C for 30 s, 55°C for 30 s, and 72°C for 45 s; and a final extension at 72°C for 10 min. PCR products were examined on a 2% (w/v) agarose gel and purified using the QIAquick Gel Extraction Kit (Qiagen, Valencia, CA, United States). The purified products were then quantified using a Qubit 2.0 Fluorometer (Invitrogen, Carlsbad, CA, United States), pooled in equimolar amounts, and sequenced as paired-end reads on an Illumina MiSeq platform according to standard protocols.

### Enrichment and Isolation of Phenol-Degrading Bacteria

In order to isolate phenol-degrading bacteria, microcosm samples were prepared from carbon–sand samples. Briefly, 10 g of the carbon–sand sample was put into an Erlenmeyer flask containing 80 mL sterilized phosphate-buffered saline (PBS). Then, the Erlenmeyer flask was placed in an ultrasonic cleaner to extract bacteria from the carbon–sand sample into the PBS solution. Next, 1 mL of supernatant was added to 100 mL of mineral salt medium (MSM) supplemented with 50 mg/L phenol as the sole carbon source. The trace element composition of the MSM was as follows (mg L^−1^): 0.2 MnCl_2_⋅4H_2_O, 0.2 CuCl_2_⋅2H_2_O, 0.2 ZnSO_4_⋅7H_2_O, 0.3 H_3_BO_3_, 0.4 CoCl_2_⋅6H_2_O, 0.4 FeCl_3_⋅6H_2_O, and 2 Na_2_MoO_4_⋅2H_2_O. The major element composition was as follows (g L^−1^): 0.05 CaSO_4_⋅2H_2_O, 2 NaHPO_4_⋅12H_2_O, 0.7 KH_2_PO_4_, 0.5 NH_4_Cl, 0.2 NaCl, and 0.1 MgSO_4_⋅7H_2_O. Subsequently, the enrichment flask was incubated at 30°C and shaken at 120 rpm. The medium was renewed at 72-h intervals. This process was repeated three times. Strains with different morphologies were isolated and continuously purified on new MSM plates containing 50 mg/L phenol until pure colonies were obtained.

Subsequently, the phenol degradation capacities of the isolates were evaluated. Isolates were firstly incubated in an R2A fluid nutrient medium for 24 h, and then the cell suspensions were pelleted by centrifugation at 5,000 × *g* for 10 min. The supernatant was then discarded. Sterilized PBS solution was used to wash the cells, which were then re-suspended in a flask containing 80 mL of MSM supplemented with 500 μg/L phenol as a carbon and energy source. Samples of the MSM solution were collected at appropriate intervals for phenol quantification. The determination of phenol concentration was performed as in a previous study (Gu et al., 2016).

For the identification of phenol-degrading bacteria, DNA was extracted using a bacterial DNA extraction kit (Dongshen Biotech, Guangzhou, China) according to the manufacturer’s instructions. The 16S rRNA gene was amplified using bacterial universal primers 27f (5′-GTGCTGCAGAGAGTTTGATCCTGGCTCAG-3′) and 1492r (5′-CACGGATCCTACGGGTACCTTGTTACGACTT-3′). The amplification reaction was performed in a 25-μL volume containing 1 μL each primer, 12.5 μL Green Master Mix (Takara, Dalian, China), 8.5 μL nuclease-free water, and 2 μL DNA template. An initial denaturation step of 5 min at 94°C was conducted, followed by 35 cycles of 94°C for 30 s, 55°C for 45 s, and 72°C for 90 s. The procedure was completed with a final elongation step at 72°C for 10 min. PCR products were sequenced (Sangon, Shanghai, China), and the sequences were compared with the bacterial 16S rDNA sequences in GenBank using the National Center for Biotechnology Information (NCBI) Basic Local Alignment Search Tool (BLAST) program. Neighbor-joining phylogenetic trees were constructed using the Molecular Evolutionary Genetics Analysis (MEGA) program version 6. The reliability of phylogenetic reconstruction was estimated using bootstrap analysis (1000 replicates).

In order to observe the morphology of effective phenol-degrading bacteria. The cells of effective phenol-degrading bacteria grown in MSM amended phenol as sole carbon and energy source for 12 h in Erlenmeyer flask, when the cells grew to the exponential phase, several drops of cell suspension were settled onto a collodion-coated copper grid of mesh size 200 for 5 min. After a brief wash with water, the grid was immersed in 30 μL of filtered 2% phosphotungstic acid (pH 7.0) or 5 mg of ruthenium red/mL for 3 min. The grid was removed, and the excess stain was wicked away with filter paper. The grid was then washed with water and dried at room temperature for 45 min. The specimens were observed under an H7500 TEM (Hitachi, Tokyo, Japan) operating at 80 kV.

### Immobilization of *Rhodococcus* sp. CS-1 Cells

Prepared sterilized polyhedral hollow polypropylene balls were placed into a 500-mL flask containing 200 mL of MSM supplemented with 4 mM phenol as the sole carbon and energy source. Then, this mixture was incubated without shaking at 30°C to allow *Rhodococcus* sp. CS-1 cells to attach to the polyhedral hollow polypropylene balls, samples of which were removed at 24, 48, 72, 96, and 120 h with sterile forceps. Subsequently, polyhedral hollow polypropylene balls were rinsed with sterile PBS solution to remove any *Rhodococcus* sp. CS-1 cells that were not sufficiently adhered. Then, the rinsed polyhedral hollow polypropylene balls were analyzed via scanning electron microscopy (SEM) and confocal laser scanning microscopy (CLSM), as described below.

### Detection of Biofilm by Scanning Electron Microscopy and Confocal Laser Scanning Microscopy

*Rhodococcus* sp. CS-1 cells immobilized on polyhedral hollow polypropylene balls were examined by SEM (S-3000N, Hitachi, Tokyo, Japan). For the preparations, cells were fixed in 0.1 M PBS containing 3% (v/v) glutaraldehyde at 4°C for 5 h. The samples were then washed with PBS (pH 7.0) six times (20 min each). For dehydration, samples were treated twice with an ethanol series of 30 and 50% for 10 min each, followed by 70, 90, and 100% (v/v) ethanol for 20 min each. Next, tert-butanol was used in place of ethanol twice for 20 min each. Dehydrated cells were filtered through a 0.2-μM polycarbonate filter, dried with a CO_2_-critical point dryer, coated with gold, and observed subsequently by SEM at 20 kV. CLSM (Zeiss, Berlin, Germany) analysis was performed using a LIVE/DEAD^®^ BacLight^TM^ Bacterial Viability and Counting Kit, including PI and SYTO9.

### Adennosine Tri-phosphate (ATP) Analysis

Adennosine tri-phosphate measurements were taken to estimate the active biomass of *Rhodococcus* sp. CS-1 immobilized on the polyhedral hollow polypropylene balls. For the preparations, polyhedral hollow polypropylene balls were removed from the incubation flask and then rinsed with PBS three times in order to remove unattached cells. Subsequently, the rinsed polyhedral hollow polypropylene balls with PBS were placed into an ultrasonic cleaner and shaken for 5 min. The supernatant was used for ATP measurement, as described in a previous study (Zhang et al., 2011).

### Phenol Biodegradation Test by Immobilized Rhodococcus sp. CS-1 Cells

Six glass columns (inner diameter = 40 cm) with a working volume of 450 mL were filled with polyhedral hollow polypropylene balls that had been previously sterilized. Three columns of polyhedral hollow polypropylene balls were used as controls, and the other three samples contained immobilized *Rhodococcus* sp. CS-1. Sand-filtered water samples with different initial phenol concentrations of 50, 250, or 500 μg/L were assessed using peristaltic pumps (Lange, Beijing, China), and the empty bed contact time (EBCT) was 18 min. The entire water treatment process was maintained at room temperature. Effluent water samples with different initial phenol concentrations were collected after 1 h for phenol detection.

### Bacteria Strains and Cultivation

*Rhodococcus* sp. CS-1 cells were inoculated into R2A fluid nutrient medium and incubated until the culture reached exponential phase; then, 200 μL of R2A pre-culture was inoculated into an Erlenmeyer flask (1000 mL) containing MSM supplemented with glucose (4 mM) as the sole carbon source, and this was defined as the control (CK). The composition of MSM was as follows (mg L^−1^): 0.1 MgSO_4_⋅7H_2_O, 0.2 NaCl, 0.5 NH_4_Cl, 0.5 Na_2_HPO_4_⋅12H_2_O, 0.5 KH_2_PO_4_, 0.1 FeCl_3_⋅6H2O, and 0.1 CaSO_4_⋅H_2_O. Cultures were incubated at 30°C and shaken at 120 rpm in an environmental chamber until reaching the exponential growth phase. In addition, Erlenmeyer flasks (1000 mL) containing MSM supplemented with 2 mM phenol or 4 mM phenol as the sole carbon source were defined as treatment 1 (T1) and treatment 2 (T2), respectively. During phenol biodegradation, we used an ultraviolet spectrophotometer to determine growth curves. Phenol degradation was evaluated by collecting samples of MSM every 3 h for phenol quantification. The determination of phenol concentration was performed as described previously 27.

### RNA Extraction, Library Preparation, Sequencing, and Analysis

*Rhodococcus* sp. CS-1 cells in exponential phase growing on glucose or phenol were collected and used for total RNA extraction. RNA was extracted from each sample using a MiniBEST Universal RNA Extraction Kit (TaKaRa, Dalian, China) according to the manufacturer’s instructions. The quality of the extracted RNA was assessed using an Agilent 2100 Bioanalyzer (Agilent Technologies, Santa Clara, CA, United States). The Illumina sequencing protocol was followed to generate RNA-seq reads, as in a previous study (Pei et al., 2017). Finally, the cDNA library was sequenced using an Illumina HiSeq 2000 (Illumina, San Diego, CA, United States). Sequencing reads were filtered by removing adaptor sequences and low-quality reads. High-quality reads with an overlap of a certain length were clustered together to form contigs. Functional annotation and differential expression analyses were performed as described previously (Lauritano et al., 2017).

### Validation of Differentially Expressed Genes by Quantitative PCR

After RNA extraction, complementary DNA (cDNA) was synthesized using total RNA with a PrimeScript RT reagent kit (TaKaRa, Dalian, China), and then the cDNA product was amplified by reverse transcription PCR (RT-PCR). 16sRNA was used as an internal reference in all reactions. Real-time quantification of mRNA was performed on an Eppendorf Realplex (4) PCR system (Eppendorf, Hamburg, Germany) using SYBR Premix ExTaq II kits (TaKaRa, Dalian, China). The comparative Ct method was employed for quantification of target gene expression that was normalized to 16sRNA expression and relative to the calibrator. Data were expressed as the fold change (T/CK) = 2^−ΔΔC_t_^. A list of all primers used in this study is presented in **Supplementary Table S1**.

### Enzyme Assay

When cells were grown to the late-exponential phase in MSM supplemented with 4 mM phenol, cells were harvested by centrifugation at 10,000 × *g*. Cell-free extracts were prepared as described previously (Hinteregger et al., 1992). Subsequently, the phenol hydroxylase, catechol-1,2-dioxygenase, protocatechuate-3,4-dioxygenase, and catechol-2,3-dioxygenase activity were assayed as the previous study described (Tallur et al., 2008).

## Results and Discussion

### Characterization of the Bacterial Community Structure in Carbon–Sand Filters

**FIGURE 1:**
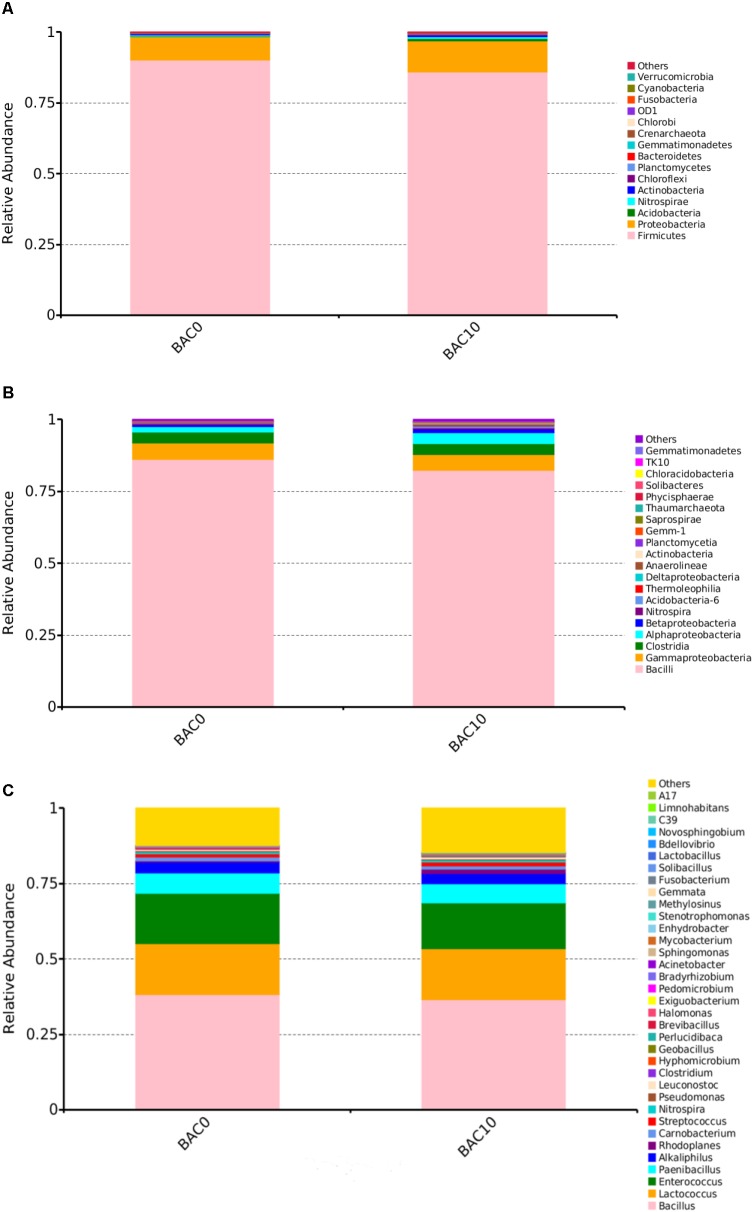
Relative abundances of bacterial community composition in the carbon–sand filters at the phylum **(A)**, class **(B)**, and genus **(C)** level. The rare species with relative abundance <0.1% are included as others.

As shown in **Figure 1A**, the most abundant bacterial phyla in the carbon–sand filters were *Firmicutes* and *Proteobacteria*. At the class level (**Figure 1B**), the predominant taxa were *Bacilli*, followed by *Gammaproteobacteria*, *Clostridia*, and *Alphaproteobacteria*. However, in some reports of BAC filter communities, the predominant classes were *Alphaproteobacteria*, *Gammaproteobacteria*, and *Acidobacteria* (Liao et al., 2012). This discrepancy may be due to differences in the time or spatial scales of sample collection (Bautista-de los Santos et al., 2016). As shown in **Figure 1C**, which shows the compositions of the two carbon–sand samples at the genus level, there were no significant differences in the bacterial communities of the carbon–sand filter at 0 and at 10 cm. The dominant bacterial genera were *Bacillus*, *Lactococcus*, and *Enterococcus*. In a previous study, we observed similar findings, as biofilms in the PE and DI pipes in a drinking water pipe network system were mainly composed of *Lactococcus* and *Escherichia–Shigella* (Zhang et al., 2017). However, we did observe that the abundances of *Bacillus* (0.38), *Enterococcus* (0.167), *Paenibacillus* (0.067), and *Alkaliphilus* (0.036) were higher in the carbon–sand filter at 0 than at 10 cm (*Bacillus* 0.37, *Enterococcus* 0.151, *Paenibacillus* 0.064, and *Alkaliphilus* 0.034). Many studies have shown that the microbial community structure is closely related to various environmental factors, such as levels of DO (De et al., 2005), nutrients (Lindström, 2000), and organic matter (Crump et al., 2003). In a drinking water biofilter, ammonia, organic matter, and DO are gradually consumed by biofilms as the depth of the filter increases. The amount of DO at a depth of 0 cm in a carbon–sand layer is known to be higher than that at a depth of 10 cm. Moreover, the adsorption capacity of organic compounds at 0 cm in a carbon–sand layer is also higher than that at 10 cm (Madoni et al., 2001). *Bacillus* and *Enterococcus* are aerobic bacteria; therefore, the microbial community structures observed at a depth of 0 and at a depth of 10 cm in a carbon–sand layer are dependent on the DO and organic matter content. Previous studies have shown that *Bacillus* can degrade many types of organic compounds, such as naphthalene (Lin et al., 2010), crude oil (Thavasi et al., 2011), and methylene blue (Chen et al., 2015). What’s more, *Bacillus* were also reported to degrade phenol (Banerjee and Ghoshal, 2010). *Lactococcus* are effective degraders of bisphenol A (Endo et al., 2007), but there are no reports that *Lactococcus* could degrade phenol. Similarly, *Enterococcus* could degrade dye (Joutey et al., 2013), but there were no informantion on its phenol degradation. Besides the dominant bacterial genera, other genera also have the potential to remove organic pollutants, including phenol. For instance, *Pseudomonas* contributes to the biodegradation of 2,4-dinitrotoluene (Spanggord et al., 1991), toluene (Reardon et al., 2000), and petroleum hydrocarbons (Varjani and Upasani, 2016). Furthermore, it reports that *Pseudomonas* always performed well in phenol biodegradation (Bertollo et al., 2017). Previous data shown demonstrated that *Rhodoplanes* is an aromatic degrader of PAHs and phenol (Ailijiang et al., 2016). In addition, streptococcus could also degrade phenol (Mohite et al., 2010). Therefore, the microorganisms in carbon–sand filters have the potential to remove organic matter, including phenol. Thus, carbon–sand filters are an ideal location for isolating a microorganism or a consortium of microorganisms capable of degrading phenol, which is of practical importance.

### Enrichment and Isolation of Phenol-Degrading Bacteria

**FIGURE 2:**
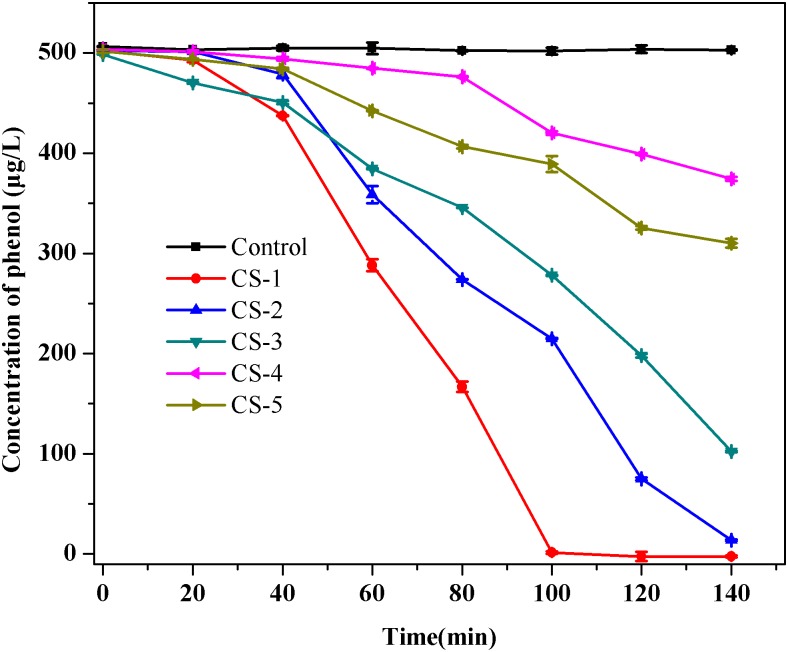
Phenol biodegradation by the five isolates CS-1, CS-2, CS-3, CS-4, and CS-5. Cultures were incubated in 80 mL of MSM supplemented with 500 μg/L of phenol at 30°C at 120 rpm. No phenol degradation occurred in the autoclaved control bottles. The mean values from triplicate experiments and the standard errors of the means, indicated by error bars, are shown.

Five isolates were obtained after phenol enrichment and are referred to as strains CS-1, CS-2, CS-3, CS-4, and CS-5, respectively. Subsequently, in order to investigate the low-concentration of phenol removing ability by indigenous bacteria, which can predict their phenol repair capability in drinking water source. A phenol degradation test of the five isolates (**Figure 2**) showed that strain CS-1 possessed the strongest ability to degrade phenol, degrading 500 μg/L phenol within 100 min. Thus, strain CS-1 was selected for further study.

The 16S rDNA sequence of strain CS-1 was compared with the bacterial 16S rDNA sequences in GenBank. The results showed that strain CS-1 was 99% similar to *Rhodococcus pyridinivorans* SB3094. As shown in **Supplementary Figure S1a**, the phylogenetic tree indicates that strain CS-1 is closely related to members of the genus *Rhodococcus.* Meanwhile, the 16S rDNA sequence of the phenol-degrading *Rhodococcus* sp. CS-1 strain was deposited in GenBank under accession number MH254944. In addition, based on the results of TEM (**Supplementary Figure S1b**), strain CS-1 is with a cell diameter ranging from 0.5 to 0.6 μm and length ranging from 0.8 to 1.5 μm. Strain CS-1 does not have any flagella. To our knowledge, *Rhodococcus* spp. are reported to degrade many kinds of organic compounds, such as oestrogens (Yoshimoto et al., 2004), polyethylene (Orr et al., 2004), and phenol (Margesin et al., 2005). Therefore, *Rhodococcus* sp. CS-1 has the potential for environmental remediation.

### Detection of Biofilm by Scanning Electron Microscopy and Confocal Laser Scanning Microscopy

**FIGURE 3:**
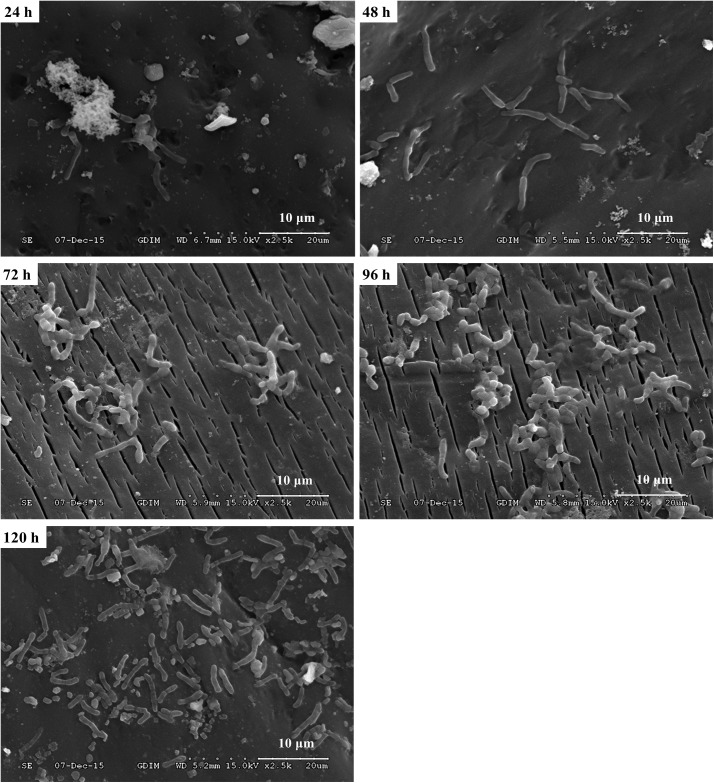
Growth of a *Rhodococcus* sp. CS-1 biofilm on polyhedron hollow polypropylene balls visualized by scanning electron microscopy.

**FIGURE 4:**
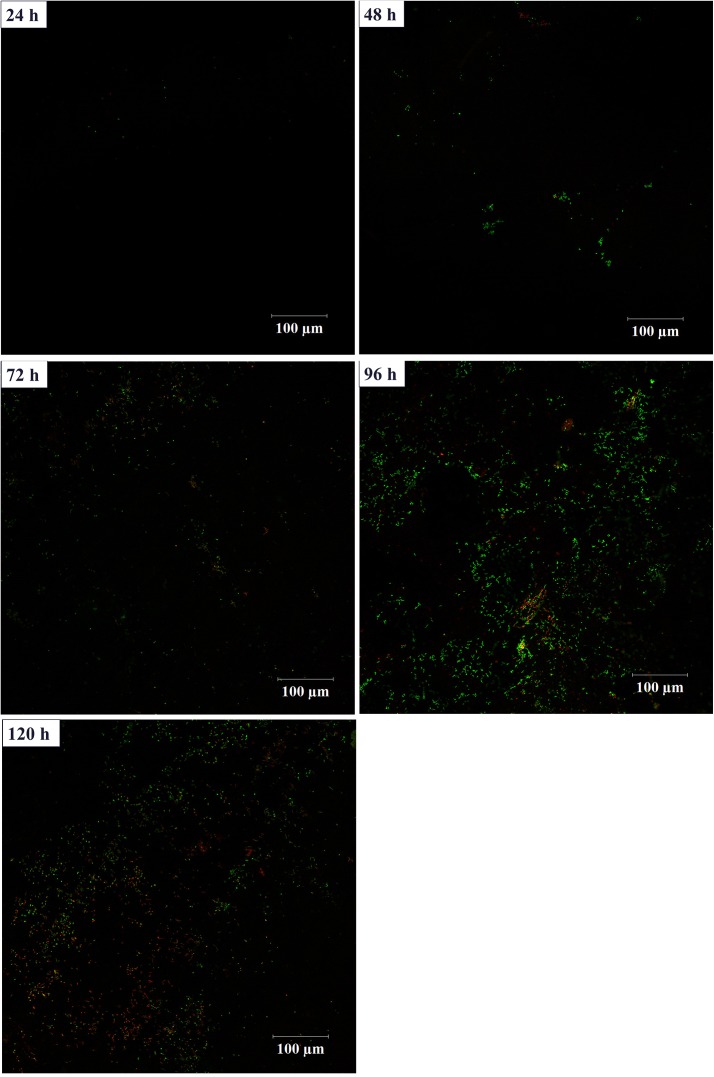
Growth of a *Rhodococcus* sp. CS-1 biofilm on polyhedron hollow polypropylene balls visualized by confocal laser scanning microscopy. Live bacteria are green and dead cells are visualized in red.

In order to investigate the biofilm-forming ability of *Rhodococcus* sp. CS-1, we used SEM and CLSM to observe. SEM visualization demonstrated that *Rhodococcus* sp. CS-1 successfully attached to and grew well on polyhedral hollow polypropylene balls (**Figure 3**). The surfaces of the polyhedral hollow polypropylene balls were covered with the greatest number of *Rhodococcus* sp. CS-1 cells after 120 h of immobilization. CLSM confirmed that the quantity of *Rhodococcus* sp. CS-1 cells on the polypropylene balls increased with the extension of the cultivation time. However, dead cells (red spots) also gradually increased in number (**Figure 4**). These results were in agreement with those obtained by measuring the ATP concentration, which demonstrated that the highest biological activity of immobilized *Rhodococcus* sp. CS-1 cells was derived from those cultivated for 96 h and that the biological activity decreased when cells were grown for 120 h (**Table 1**). These results indicate that the *Rhodococcus* sp. CS-1 biofilm reached an optimal state after 96 h of cultivation. Therefore, we selected this state for the assessment of phenol biodegradation by the attached biomass, which could shorten run-up time for phenol biodegradation.

**Table 1:** Adenosine tri-phosphate (ATP) analysis of *Rhodococcus* sp. CS-1 immobilized on polyhedron hollow polypropylene balls at different times.

Time (h)	24	48	72	96	120
ATP (mol/g)	(0.98 ± 0.53) × 10^−8^	(1.21 ± 0.31) × 10^−8^	(1.52 ± 0.37) × 10^−8^	(3.69 ± 0.19) × 10^−8^	(0.88 ± 0.71) × 10^−8^

### Phenol Biodegradation Test by Immobilized *Rhodococcus* sp. CS-1 Cells

When *Rhodococcus* sp. CS-1 was immobilized on sterilized polyhedral hollow polypropylene balls, it still exhibited a strong ability to remove phenol. Phenol removal reached approximately 12% with an initial phenol concentration of 500 μg/L and increased to approximately 36% with an initial phenol concentration of 50 μg/L, as shown in **Supplementary Figure S2**. All the results indicated that immobilized *Rhodococcus* sp. CS-1 cells have the potential to use in phenol actual water treatment.

### Bacteria Strains and Cultivation

**FIGURE 5:**
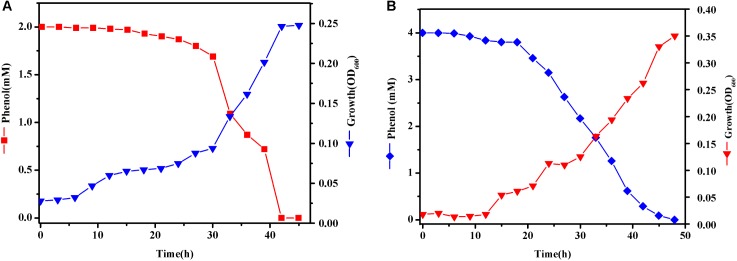
Growth curves of *Rhodococcus* sp. CS-1. Cells were grown at 30°C in MSM with 2 **(A)** and 4 mM **(B)** phenol as the sole carbon and energy source. Growth was followed using spectrophotometer method.

In order to get more differentially expressed genes, we chose a higher phenol concentration of 2 and 4 mM to determine which genes are specifically expressed during phenol biodegradation. Cells were treated with various concentrations of phenol and harvested when the phenol biodegradation rate reached its peak. When cells were grown in MSM with 2 mM phenol (T1), cells were harvested at 33 h (**Figure 5A**). These cells grew quickly at log-phase, which was accompanied by a dramatic decrease in the phenol concentration at 33 h, indicating that the bacteria were using phenol as the sole carbon and energy source. Similarly, when cells were grown in MSM with 4 mM phenol (T2), cells were harvested at 33 h (**Figure 5B**).

### Transcriptome Sequences Assembly and Analysis

#### Transcriptome Sequences Assembly, GOC, and GO Analysis

To investigate the phenol biodegradation mechanisms of *Rhodococcus* sp. CS-1 further, we employed a comparative transcriptomic approach by sequencing total RNA from CK, T1, and T2 samples. High-throughput RNA-seq generated 1,434,430,365 and 1,568,761,089 bp of clean reads from control samples CK-1 and CK-2, respectively. Similarly, 1,510,266,961 and 1,352,793,573 bp of clean reads were obtained from treatment groups T1-1 and T1-2, respectively, and 1,493,601,571 and 1,439,138,557 bp of clean reads were obtained from treatment groups T2-1 and T2-2, respectively. The Q20 of all samples was over 96%, and the Q30 was over 91%, indicating that the clean reads were of good quality. In total, 2,315 genes were identified, providing abundant data for the analysis of phenol biodegradation by *Rhodococcus* sp. CS-1.

All identified unigenes were divided into 21 clusters according to clusters of orthologous genes (COG) annotation (**Supplementary Figure S3**). The largest of the five clusters was the R cluster (general function prediction only), followed by the E (amino acid transport and metabolism), I (lipid transport and metabolism), C (energy production and conversion), and Q (secondary metabolites biosynthesis, transport, and catabolism) clusters.

According to Gene Ontology (GO) analysis, 1,780 unigenes were classified into the three GO categories of cellular component, biological process, and molecular function (**Supplementary Figure S3**). The subcategories with the highest unigene representation were catalytic activity (1,382 unigenes), followed by metabolic process (1,229 unigenes), binding (987 unigenes), cellular process (926 unigenes), single-organism process (875 unigenes), cell (290 unigenes), and cell part (290 unigenes).

#### KEGG Enrichment and Candidate DEGs Analysis Involves in Phenol Biodegradation

**FIGURE 6:**
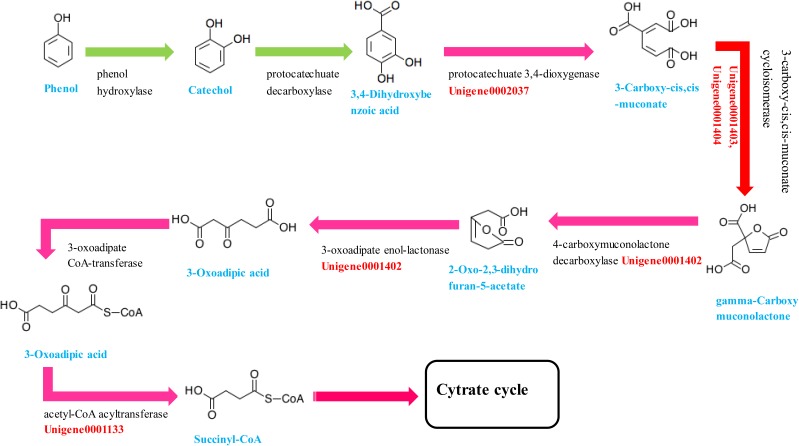
The protocatechuate branch of the β-ketoadipate pathway in *Rhodococcus* sp. CS-1 during phenol biodegradation.

KEGG enrichment analysis demonstrated that carbon metabolism (16.14%), biosynthesis of amino acids (14.25%), pyruvate metabolism (7.18%), purine metabolism (6.95%), glyoxylate and dicarboxylate metabolism (6.95%), and fatty acid metabolism (6.6%) were the most significantly enriched. This finding indicates that the majority of annotated genes are involved in the basal metabolism of *Rhodococcus* sp. CS-1, providing the molecules and energy necessary for growth. Interestingly, genes involved in the degradation of aromatic compounds (4.97%) and benzoate degradation (4.3%) were also successfully enriched among the expressed genes. According to the KEGG database, the phenol degradation pathway is a part of the benzoate degradation pathway, thus indicating the capacity for phenol degradation in *Rhodococcus* sp. CS-1. A list of the unigenes involved in the aromatic compounds and benzoate degradation pathways is provided in **Table 2**. The major metabolic pathways of phenol involve the β-ketoadipate and meta-cleavage pathways (Mahiudddin et al., 2012). Previous studies have shown that the β-ketoadipate pathway consists of two parallel branches in which the aromatic rings are cleaved by either protocatechuate 3,4-dioxygenase or catechol 1,2-dioxygenase. Although the protocatechuate branch of the β-ketoadipate pathway has been widely studied in Gram-negative bacteria (Shen and Liu, 2005), this pathway has been poorly investigated in Gram-positive bacteria. Some researchers have found that *Corynebacterium glutamicum* does not exhibit significant protocatechuate 3,4-dioxygenase activity when cultivated with phenol (Shen and Liu, 2005). In this study, we found that protocatechuate 3,4-dioxygenase was down-regulated and 3-carboxy-*cis*,*cis*-muconate cycloisomerase, 4-carboxymuconolactone decarboxylase, and 3-oxoadipate enol-lactonase were up-regulated, not only cultured in 2 mM phenol but also in 4 mM phenol compared with control groups. In addition, a previous study found that the protocatechuate branch of the β-ketoadipate pathway was used by *Arthrobacter* for phenol degradation, as protocatechuate 3,4-dioxygenase, 3-carboxy-*cis*,*cis*-muconate cycloisomerase, and 4-carboxymuconolactone decarboxylase were up-regulated during phenol degradation (Li et al., 2016). Similarly, when *Pseudomonas putida* KT2440 is cultured in medium with benzoate as the sole carbon source, the protocatechuate branch of the β-ketoadipate pathway was found to be activated using the 1-DE MudPIT method (Yun et al., 2011). In this study, *Rhodococcus* sp. CS-1 was also found to use the protocatechuate branch of the β-ketoadipate pathway, as shown in **Figure 6**. Moreover, it appears to also use the catechol branch of the β-ketoadipate pathway to degrade phenol, as the key enzyme in this pathway, catechol 1,2-dioxygenase, was significantly up-regulated (**Table 2**).

**Table 2:** Functions of key genes detected by RNA-Seq.

Gene ID	Description	COG No.	KEGG No.	EC No.	Predicted function	Fold changes [log2 Ratio(T1/CK)]	Fold changes [log2 Ratio(T2/CK)]
**Up-regulated genes**							
Unigene0001554	Copper oxidase	COG2132	NA	NA	Transition metal ion binding	7.931463625	7.587918713
Unigene0001727	4-Hydroxyphenylacetate 3-monooxygenase	COG2368	K00483	[EC:1.14.14.9]	Oxidoreductase activity	7.05948939	7.567045946
Unigene0000462	Copper chaperone	COG2608	NA	NA	Metal ion binding	6.281616947	6.145808165
Unigene0001819	Cell wall-associated hydrolase	NA	NA	NA	Catalytic activity	6.20687524	6.286267865
Unigene0001442	Catechol 1,2-dioxygenase	COG3485	K03381	[EC:1.13.11.1]	Iron ion binding	4.561407055	4.913769773
Unigene0001044	3-Isopropylmalate dehydratase small subunit, partial	COG0066	K01704	[EC:4.2.1.334.2.1.35]	Biosynthesis of amino acids	4.239888909	5.08225346
Unigene0001362	Porin	NA	NA	NA	Cellular process	3.993185145	2.749292698
Unigene0001402	3-Oxoadipate enol-lactonase/4-carboxymuconolactone decarboxylase	COG1414	K14727	[EC:3.1.1.24 4.1.1.44]	Cellular aromatic compound metabolic process	3.566354186	3.952037315
Unigene0000868	ATP synthase subunit beta	COG0055	K02112	[EC:3.6.3.14]	Cellular process	3.316156883	1.980735278
Unigene0000870	ATP synthase A chain		K02108		Single-organism process	3.23418115	NA
Unigene0000595	MarR family transcriptional regulator	COG1846	NA	NA	Regulation of biological process	3.028816138	4.047003968
Unigene0002230	MarR family transcriptional regulator	COG2390	K05346	NA	Organic cyclic compound binding	2.788254279	2.919634293
Unigene0001015	GntR family transcriptional regulator	NA	NA	NA	Regulation of metabolic process	2.613034352	4.602239966
Unigene0001500	Membrane protein	NA	NA	NA	NA	2.49129552	3.21933403
Unigene0001729	AraC family transcriptional regulator	COG2207	NA	NA	Regulation of biological process	2.447191904	1.997520806
Unigene0001404	3-Carboxy-*cis*,*cis*-muconate cycloisomerase	NA	K01857	[EC:5.5.1.2]	Aromatic compound catabolic	2.075903663	2.466726915
Unigene0000594	4-Oxalocrotonate tautomerase	COG1942	K01821	[EC:5.3.2.6]	Catalytic activity	2.075329564	2.862102576
Unigene0001403	3-Carboxy-*cis*,*cis*-muconate cycloisomerase	COG0015	K01857	[EC:5.5.1.2]	Transferase activity	2.070413068	NA
Unigene0000594	4-Oxalocrotonate tautomerase	COG1942	K01821	[EC:5.3.2.6]	Degradation of aromatic	2.075329564	2.862102576
Unigene0001077	Membrane protein	NA	NA	NA	Nucleic acid binding transcription factor	1.947384027	3.254483446
Unigene0002212	Integral membrane protein	NA	NA	NA	NA	1.879861463	1.99330912
Unigene0001698	Enoyl-CoA hydratase	COG1024	K01692	[EC:4.2.1.17]	Catalytic activity	1.861549387	NA
Unigene0001846	3-Hydroxyacyl-CoA dehydrogenase	COG1250	K01782	[EC:1.1.1.35 4.2.1.17 5.1.2.3]	Oxidoreductase activity	1.302542598	2.208439432
**Down-regulated genes**							
Unigene0000009	Photosystem II q(b) protein	NA	K02703	NA	Protein modification process	−15.27605896	−7.072221575
Unigene0001722	Benzoate/toluate 1,2-dioxygenase subunit alpha	COG4638	K05549	[EC:1.18.1.-]	Iron-sulfur cluster binding	−3.246405396	−3.136857792
Unigene0002041	Cell division/cell wall cluster transcriptional repressor MraZ	COG2001	K03925	NA	Biological regulation	−2.182189181	−1.469538639
Unigene0000852	DeoR family transcripitonal regulator	COG3618	NA	NA	Biological regulation	−2.049113817	−1.441305159
Unigene0000975	Two-component sensor histidine kinase	COG1024	K01692	[EC:4.2.1.17]	Transferase activity	−1.947578691	NA
Unigene0000270	LuxR family transcriptional regulator	COG0277	NA	NA	Biological regulation	−1.667626132	−3.350484629
Unigene0000525	Acetyl-CoA *C*-acetyltransferase	COG0183	K00626	[EC:2.3.1.9]	Transferase activity	−1.641201222	−2.073170481
Unigene0001651	TetR family transcriptional regulator	COG0736	NA	NA	Organic cyclic compound binding	−1.566589349	−1.63502311890494
Unigene0002037	Protocatechuate 3,4-dioxygenase, beta subunit	COG0266	K10563	[EC:1.13.11.3]	Deoxyribonuclease activity	−1.391999616	−1.534084928
Unigene0001839	4-Hydroxy-2-oxovalerate aldolase	COG0119	K01666	[EC:4.1.3.39]	Cation binding	−1.362872634	NA
Unigene0001308	Helix-turn-helix transcriptional regulator	COG1741	K06911	NA	Nucleic acid binding transcription factor activity	−1.174867987	NA

*N/A, not available.*

Comparison of the CK group and T1 group revealed a total of 302 differentially expressed genes (DEGs) annotated by the KEGG database. Biosynthesis of amino acids and carbon metabolism was the most significantly enriched functions at 19.5 and 18.9%, respectively. A total of 15% of unigenes were involved in the degradation of aromatic compounds, and 13% of unigenes were involved in the benzoate pathway. Comparison of the CK group and T2 group revealed that there were 244 DEGs annotated by the KEGG database. Similarly, genes involved in carbon metabolism and the biosynthesis of amino acids accounted for 20 and 16% of the genes, respectively, and were the most significantly enriched pathways. In addition, 12% of unigenes were involved in the degradation of aromatic compounds, and 10% of unigenes were involved in the benzoate pathway. These findings indicate that *Rhodococcus* sp. CS-1 has the ability to degrade phenol.

Except for KEGG enrichment key genes, some genes such as regulatory factors were important during phenol biodegradation (**Table 2**). Previous literature indicated that the LuxR-type regulators are generally functioning as transcriptional activators and control a wide variety of functions in various biological processes, for instance in biofilm and spore formation, cell division, plasmid transfer, and bacterial virulence (Hansmeier et al., 2006). However, in this study, the expression of LuxR-type regulators was down-regulated during phenol biodegradation. The cause of this phenomenon needs further analysis. We can see that the expression of AraC family transcriptional regulator in medium supplemented with phenol was up-regulated. The result was consistent with the report that the AraC family of transcriptional regulators usually activate transcription through totally different mechanisms (Ramos et al., 1990). In this study, we found that MarR family transcriptional regulator was significantly up-regulated. MarR family transcriptional regulator was reported to play important roles in antibiotic resistance, virulence, and catabolism (Davis et al., 2013). Further, it has also been reported to regulate the β-ketoadipate pathway in detoxification of aromatic compounds (Fiorentino et al., 2007), and to act as a transcriptional repressor to regulate the β-ketoadipate pathway for aromatic catabolism (Davis et al., 2013). But the function of the MarR family transcriptional regulator in *Rhodococcus* CS-1 during phenol biodegradation needs to be further investigated. There was little information about the DeoR family transcriptional regulator involved in aromatic compounds. DeoR family transcripitonal regulator was reported involving in the transcriptional regulation of the fructose-PTS and acting as a repressor (Gaigalat et al., 2007). In this study, we found that DeoR family transcripitonal regulator was down-regulated, so it might act as a repressor during phenol biodegradation. But the specific function of DeoR family transcripitonal regulator in phenol biodegradation still need to be verified. Classically, TetR family regulators have been shown to regulate gene expression as a response to environmental stimuli (Maceachran et al., 2008). In this study, TetR family regulators might act as a repressor as its response to phenol.

Copper oxidase is reported to be able to catalyze the one-electron oxidation of phenolics, aromatic amines, and other electron-rich substrates with a concomitant reduction of O_2_ to H_2_O (Levasseur et al., 2008). In addition, other related multicopper oxidases are reported to be involved in other physiological functions including fungal development, melanin synthesis, detoxification, and human and plant pathogenesis (Levasseur et al., 2008). Copper chaperone was found up-regulated during phenol biodegradation. Previous data indicated that copper chaperone was copper-binding protein that directly inserted copper into specific targets, preventing the accumulation of free copper ions that could be toxic to the cell (Bertinato and Labbé, 2003). 3-Isopropylmalate dehydratase which was involved in branched amino acid synthesis was up-regulated (Guerreiro et al., 2010). This might be related to the rapid growth of the strain.

It is interesting that genes involved in cell membrane and wall were also activated, such as cell division/cell wall cluster transcriptional repressor MraZ (Unigene0002041), cell wall-associated hydrolase (Unigene0001819) membrane protein (Unigene0001077 and Unigene0001500), and integral membrane protein (Unigene0002212).

#### Trend Analysis of Differentially Expressed Genes (DEGs)

The expression profiles of the DEGs were illustrated by a cluster analysis by Short Time-Series Expression Miner (STEM) software. We did this to identify similar expression patterns of all DEGs across the set of stages, and how those patterns were similar or different between DEGs. Eight expression patterns (profiles) of differentially expressed genes were identified (**Supplementary Figure S4**). The most abundant group was profile 1, with 403 genes whose expression showed a negative slope when treated with phenol.These genes were expressed at their highest level at CK. Profile 0 showed continuous negative slope when treated with phenol. The second most abundant group was profile 6, which contained 208 genes that began to up-regulate at T2. Profile 2 and profile 5 consisted of 77 and 206 genes, respectively, whose expression showed a drastic changes when treated by different concentration of phenol.

#### Confirm Unigenes Expression Using Real-Time Quantitative Reverse Transcription PCR

**FIGURE 7:**
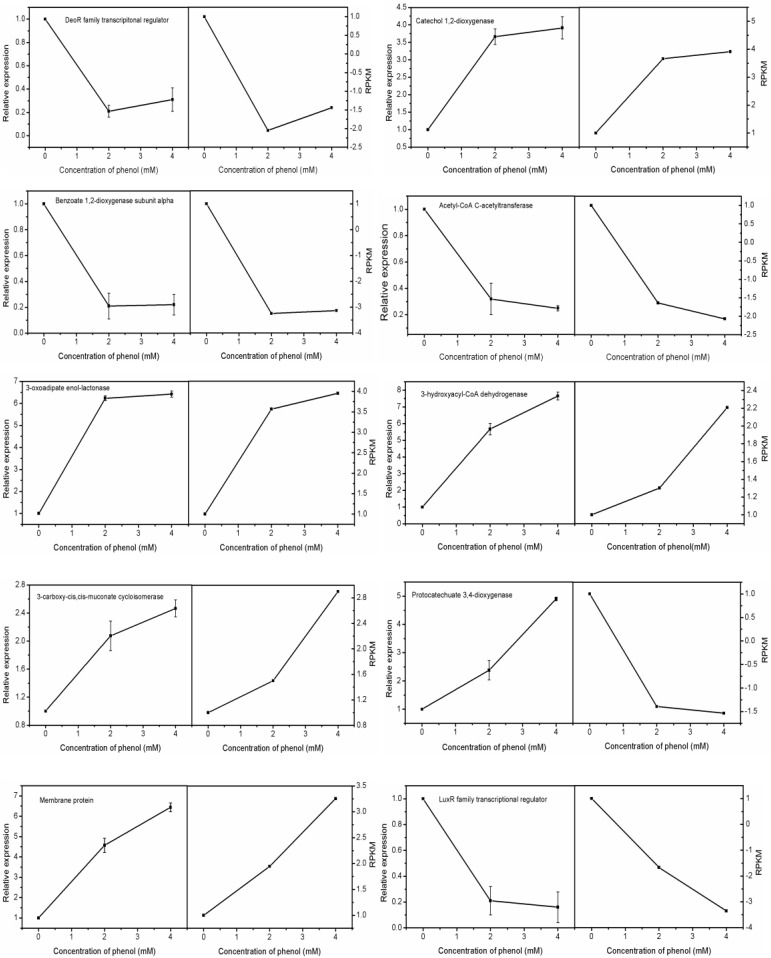
Expression levels of the candidate unigene revealed by qRT-PCR (left side) and RNA-seq (right side). Data from qRT-PCR are means of three replicates and bars represent SE.

To confirm the accuracy and reproducibility of the transcriptome analysis results, we then selected 10 candidate unigenes for real-time quantitative RT-PCR (qRT-PCR) validation. Primers of the candidate unigenes are shown in **Supplementary Materials** (**Table 1**). The log2 fold change in gene expression between control groups and treatment groups demonstrated significant correlation between RNA-seq and RT-qPCR. In addition to Unigene0002037 (protocatechuate 3,4-dioxygenase), the results for the relative expression of other genes using RNA-seq and RT-qPCR showed over 90% consistency, indicating the reliability of the RNA-seq analysis (**Figure 7**).

### Enzyme Assay

The cell-free extracts of strain CS-1 grown on phenol contained the activities of phenol hydroxylase, protocatechuate-3,4-dioxygenase, and catechol-1,2-dioxygenase but not catechol-2,3-dioxygenase (**Supplementary Table S2**). These results indicated that the enzymes of phenol degradation in both catechol and protocatechuate branch were induced. Thus, we deduced that strain CS-1 might degrade phenol via both the catechol and protocatechuate branch of the β-ketoadipate pathway.

## Conclusion

The predominant microorganisms in a carbon–sand filter are *Bacilli*, *Gammaproteobacteria*, *Clostridia*, and *Alphaproteobacteria.* A bacterial strain capable of degrading low concentrations of phenol was isolated and characterized as *Rhodococcus* sp. CS-1. Transcriptomic analysis showed that *Rhodococcus* sp. CS-1 was capable of degrading phenol via both the catechol and protocatechuate branches of the β-ketoadipate pathway. Furthermore, some novel candidate biomarkers (copper oxidase, copper chaperone, and MarR/DeoR/TetR family transcriptional regulators) were successfully identified to be potentially involved in phenol biodegradation. Therefore, strain CS-1 has strong potential for use in the bioremediation of phenol-polluted drinking water. This study provides new insights into the microbial community structure in carbon–sand filters and offers an approach for investigating the ability of native microorganisms to degrade phenol during drinking water treatment.

## Author Contributions

QW, JZ, and QG conceived and designed the experiments. QG, YZ, XW, and WG performed the experiments. JW, QG, LH, and MS analyzed the data. HW contributed reagents, materials, and analysis tools. YD, QG, and QW contributed to the writing of the manuscript.

## Conflict of Interest Statement

The authors declare that the research was conducted in the absence of any commercial or financial relationships that could be construed as a potential conflict of interest.
