# Supplementary material for: Isolation and Transcriptome Analysis of Phenol-Degrading Bacterium From Carbon–Sand Filters in a Full-Scale Drinking Water Treatment Plant
Source: Front Microbiol. 2018 Sep 21;9:2162. doi: 10.3389/fmicb.2018.02162 (PMC6160575; doi:10.3389/fmicb.2018.02162)
Supplement: Supplementary file 1 [file Table_1.DOCX]

***Supplementary Material***

**Isolation and transcriptome analysis of phenol-degrading bacterium from carbon-sand filters in a full-scale drinking water treatment plant**

Qihui Gu^1^, Qingping Wu^1^*, Jumei Zhang^1^, Weipeng Guo^1^, Yu Ding^1^, Juan Wang^1^, Xianhu Wei^1^, Youxiong Zhang^1^, Huiqing Wu^1^, Ming Sun^1^, Luanfeng Hou^1^

*** Correspondence:** Qingping Wu: E-mail: [wuqp203@163.com](mailto:wuqp203@163.com)

**Supplementary Figures**

(a)


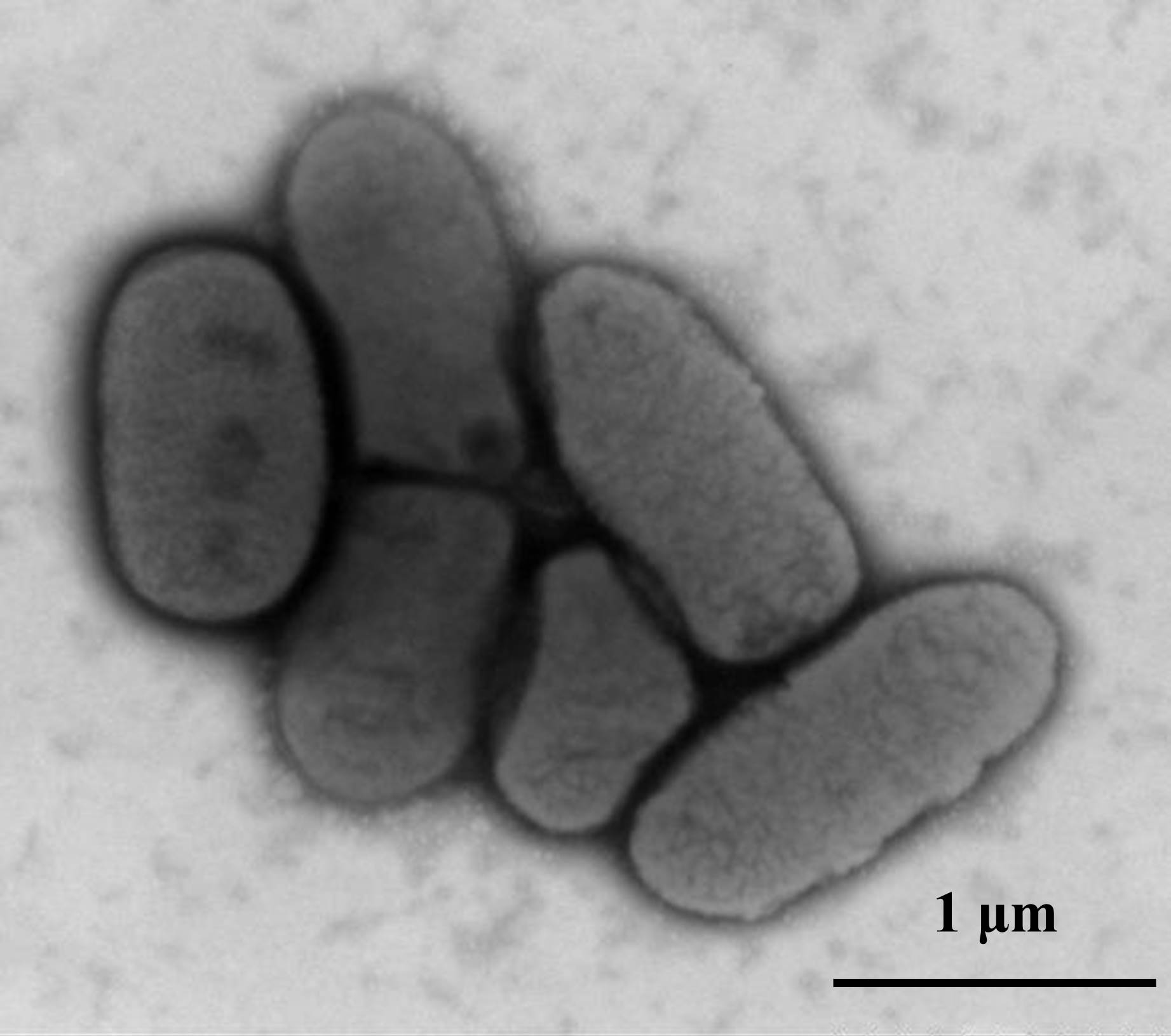


(b)

Fig.S1 (a)The neighbor-joining phylogenetic tree of 16S gene sequences was created by MEGA software 6.0. (b)Transmission electron micrograph of *Rhodococcus* sp. CS-1





Fig.S2 Phenol removal of strain CS-1 immobilized on polyhedron hollow polypropylene balls under different initial phenol concentrations. Sterilized polyhedron hollow polypropylene balls in glass column as control. The mean values from triplicate experiments and the standard errors of the means, indicated by error bars, are shown.


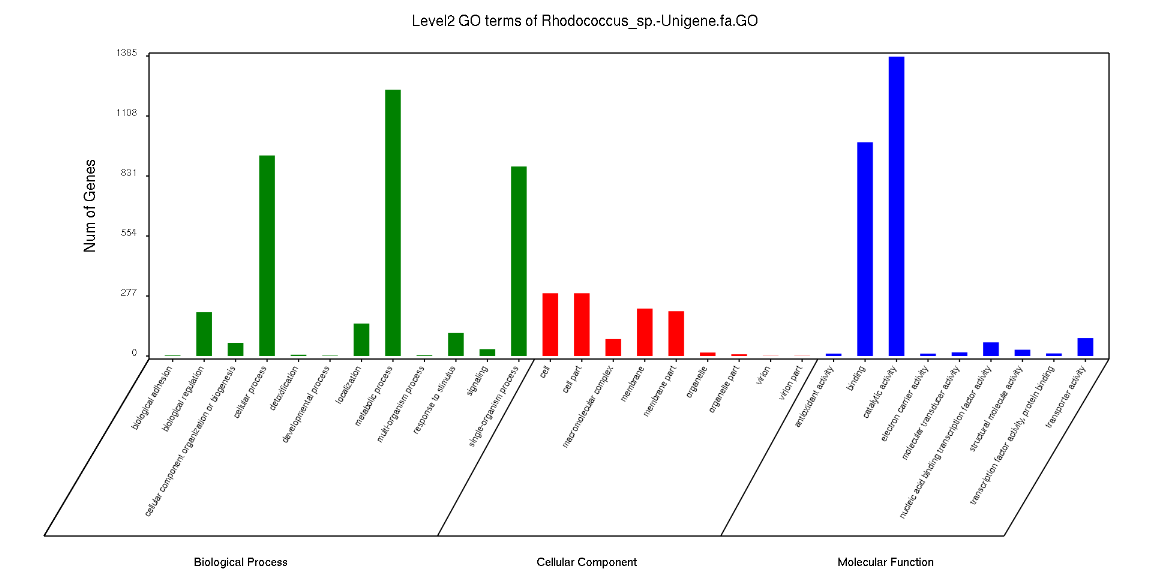


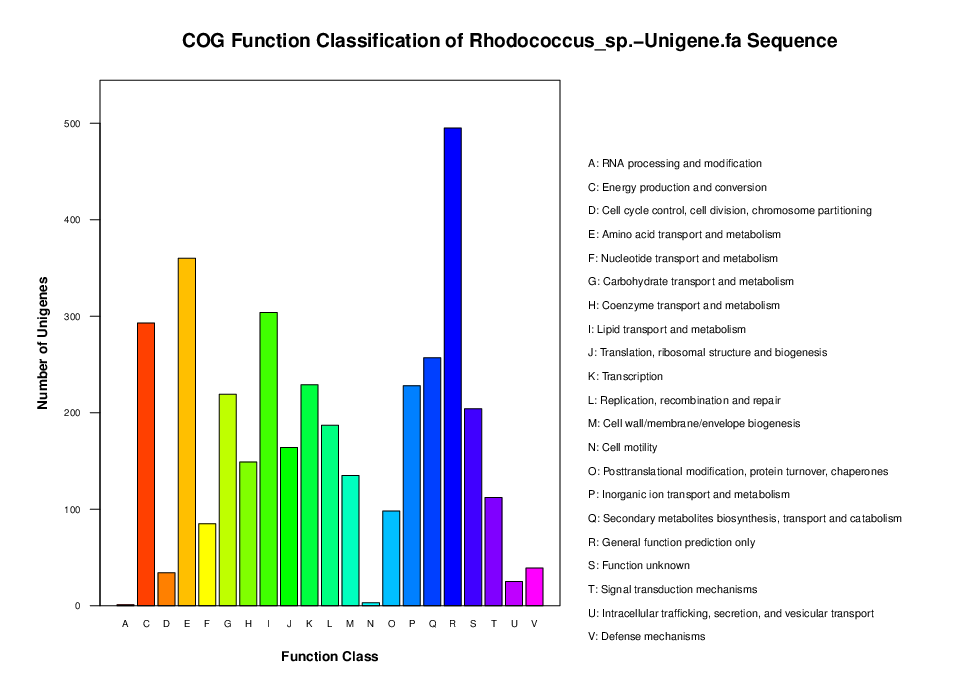


Fig.S3 GO and COG function classifications of the unigenes of CK and T samples.


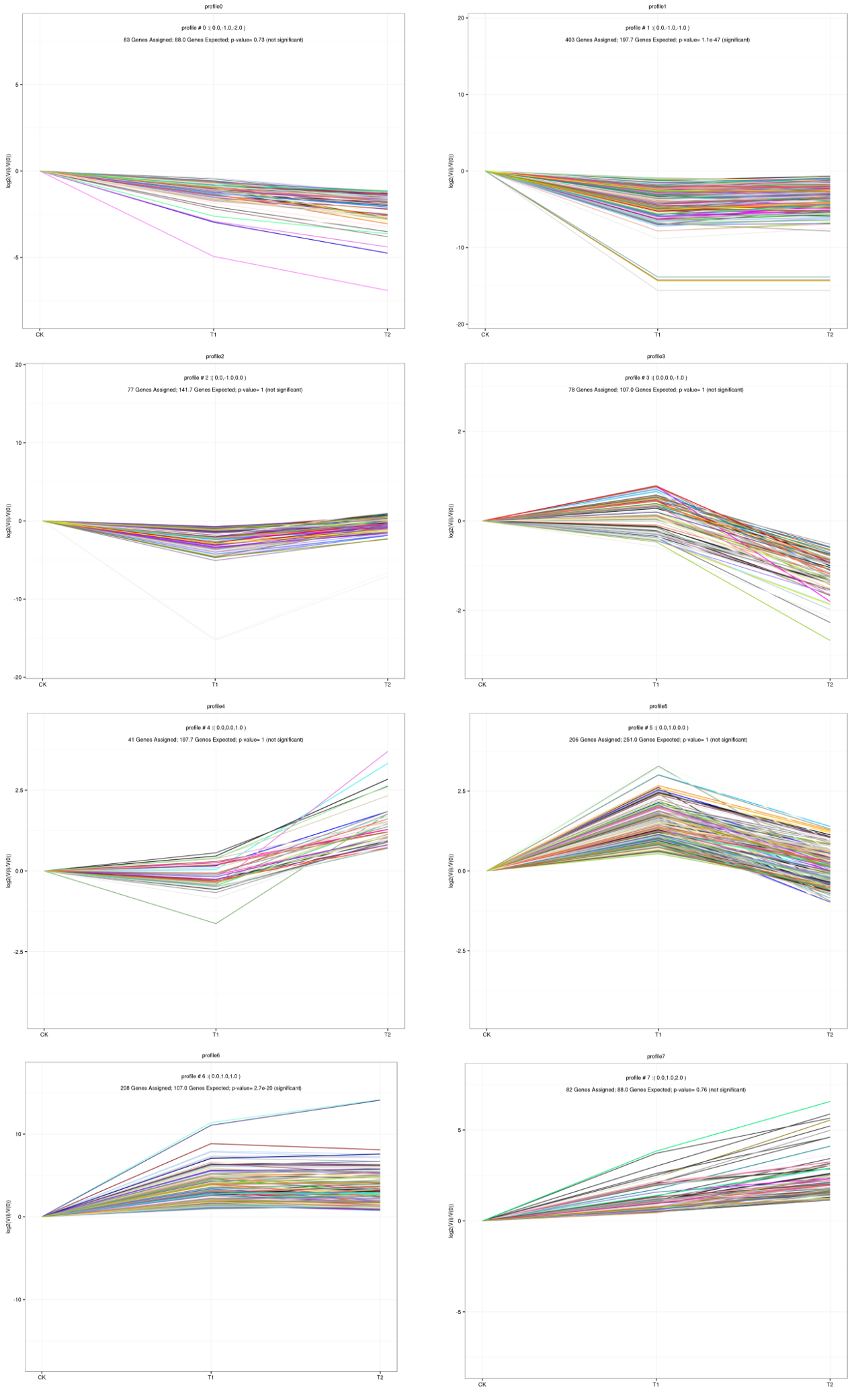

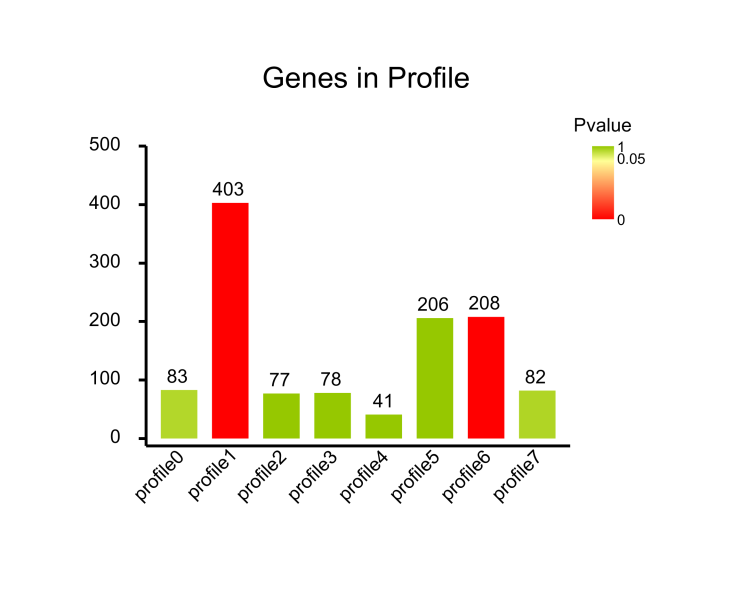


Profile7

Profile6

Profile5

Profile0

Profile4

Profile3

Profile2

Profile1

Fig.S4 DEGs expression profiles. The seven major profiles obtained by K-means algorithm, representing up-regulated (4,6,7), transient(2,5), and down-regulated (0,1,3) profile. Expression ratios are expressed as Log2.

| Table S1 Primers for RT-qPCR | | | | | |
| --- | --- | --- | --- | --- | --- |
| Seq ID | Orientation | tm | GC% | Seq length | Seq |
| Unigene0002037 | FORWARD | 60.47 | 60 | 154 | ACAGGGGTCTCTCCTCATCC |
| Unigene0002037 | REVERSE | 61.26 | 55 |  | TCGTTGAGGGTCTTCGTGAC |
| Unigene0001722 | FORWARD | 59.96 | 50 | 197 | GAACAGTTCGATGCCCGTAT |
| Unigene0001722 | REVERSE | 59.99 | 55 |  | CAGCAACGGAAGAGGAACTC |
| Unigene0001404 | FORWARD | 59.18 | 61.11 | 207 | CTGTTCGACCCCGTCTTC |
| Unigene0001404 | REVERSE | 59.55 | 64.71 |  | GGATCGTCGTCCGAGGT |
| Unigene0001402 | FORWARD | 60.08 | 50 | 181 | GGACGGATCACAAAGCTCAT |
| Unigene0001402 | REVERSE | 59.34 | 50 |  | GGGTGATGAAATCCTGGAAG |
| Unigene0000525 | FORWARD | 60.31 | 55 | 206 | GTGGAGCTCACCGAACAACT |
| Unigene0000525 | REVERSE | 60.13 | 63.16 |  | GTGGAGACCACGCCTACCT |
| Unigene0001442 | FORWARD | 59.98 | 55 | 246 | CGCTACATCGGTCCCTACAT |
| Unigene0001442 | REVERSE | 58.41 | 55 |  | GACCTCTTCGACGATCTCCT |
| Unigene0001846 | FORWARD | 60.04 | 50 | 201 | CTCAAGCACAAGGTGTTCCA |
| Unigene0001846 | REVERSE | 59.97 | 50 |  | CTCGTCGGAGGTCTTCTCAC |
| Unigene0000270 | FORWARD | 60.04 | 60 | 219 | CTACCAGCGCCTACGAGAAC |
| Unigene0000270 | REVERSE | 59.97 | 55 |  | TATCCCAGAGCGACGAGTCT |
| Unigene0001077 | FORWARD | 58.67 | 45.00 | 186 | ATGAACTCGGCAAGATGATG |
| Unigene0001077 | REVERSE | 61.15 | 63.16 |  | GAGGCACCGTCGTGGTAGT |
| Unigene0000852 | FORWARD | 59.99 | 50.00 | 162 | CGTGTTTTCGAAAGGAGAGC |
| Unigene0000852 | REVERSE | 60.41 | 52.63 |  | CAGCAAGCGTTTCCACATC |

Table S2 Specific activities of enzymes in the cell-free extract of strain CS-1 on phenol

| Enzymes | Specific activity  (Units/mg of protein) |
| --- | --- |
| Phenol hydroxylase | 0.31 |
| Catechol-1, 2-dioxygenase | 0.26 |
| Protocatechuate-3, 4-dioxygenase | 0.21 |
| Catechol- 2, 3-dioxygenase | 0.00 |
